# Gender differences in gait kinematics for patients with knee osteoarthritis

**DOI:** 10.1186/s12891-016-1013-z

**Published:** 2016-04-12

**Authors:** Angkoon Phinyomark, Sean T. Osis, Blayne A. Hettinga, Dylan Kobsar, Reed Ferber

**Affiliations:** Faculty of Kinesiology, University of Calgary, Calgary, AB Canada; Faculty of Nursing, University of Calgary, Calgary, AB Canada; Running Injury Clinic, University of Calgary, 2500 University Drive NW, Calgary, AB T2N 1N4 Canada

**Keywords:** Gait, Biomechanics, Kinematics, Knee, Osteoarthritis, Sex differences, Principal component analysis, Support vector machine

## Abstract

**Background:**

Females have a two-fold risk of developing knee osteoarthritis (OA) as compared to their male counterparts and atypical walking gait biomechanics are also considered a factor in the aetiology of knee OA. However, few studies have investigated sex-related differences in walking mechanics for patients with knee OA and of those, conflicting results have been reported. Therefore, this study was designed to examine the differences in gait kinematics (1) between male and female subjects with and without knee OA and (2) between healthy gender-matched subjects as compared with their OA counterparts.

**Methods:**

One hundred subjects with knee OA (45 males and 55 females) and 43 healthy subjects (18 males and 25 females) participated in this study. Three-dimensional kinematic data were collected during treadmill-walking and analysed using (1) a traditional approach based on discrete variables and (2) a machine learning approach based on principal component analysis (PCA) and support vector machine (SVM) using waveform data.

**Results:**

OA and healthy females exhibited significantly greater knee abduction and hip adduction angles compared to their male counterparts. No significant differences were found in any discrete gait kinematic variable between OA and healthy subjects in either the male or female group. Using PCA and SVM approaches, classification accuracies of 98–100 % were found between gender groups as well as between OA groups.

**Conclusions:**

These results suggest that care should be taken to account for gender when investigating the biomechanical aetiology of knee OA and that gender-specific analysis and rehabilitation protocols should be developed.

## Background

Osteoarthritis (OA) is the most common cause of musculoskeletal pain and disability in the knee joint and it has been suggested that disease development and progression may be related to atypical joint kinematics during gait [1]. It has also been reported that females have a two- to three-fold risk of sustaining knee OA as compared to their male counterparts [2, 3]. Amongst a large body of literature on OA gait, however, very few studies have investigated gender-related differences in walking mechanics for patients with knee OA [4–8] and of those, conflicting results have been reported. Moreover, a recent systematic review of the biomechanical variables involved in the etiology of knee OA [9] called for further investigation of gender differences in gait biomechanics in order to better understand, and thereby characterize, the unique gait patterns of older women and men and possibly detect the early changes in gait that may lead to OA pathology.

The few studies that have investigated gait differences between patients with knee OA as compared to gender-matched controls have produced conflicting results with limited sample sizes [4, 10–13]. For instance, Manetta et al. [10] investigated only male subjects and reported that sagittal plane knee range of motion (ROM) during stance was significantly reduced for OA males (*n* = 10) compared to their healthy counterparts (*n* = 10). McKean et al. [4] reported that OA females (*n* = 15) demonstrated significantly reduced sagittal plane knee ROM as compared to healthy females while OA males (*n* = 24) exhibited similar sagittal plane knee ROM as healthy males. Conversely, Ko et al. [11] reported no differences between healthy and OA subjects (*n* = 31 female; 29 male) in knee ROM irrespective of gender-specific group. Thus, there is a clear discrepancy among these aforementioned studies and further research employing larger sample sizes is needed.

There is also limited gender-specific gait research related to healthy non-OA individuals, especially in middle-aged and older adults. While increased frontal plane knee, hip and pelvis angles as well as increased transverse plane hip angles have been well documented in healthy young adult females [14–17], conflicting and limited evidence exists for their older counterparts. To our knowledge, only two studies have comprehensively investigated gender-related differences in gait kinematics for healthy older adults. Boyer et al. [18] studied 21 male and 21 female healthy adults aged 50–79 years at self-selected walking speeds and reported that healthy females exhibited a greater frontal plane hip peak adduction angle compared to males along with a reduced sagittal plane knee angle at mid-stance and a greater sagittal plane hip angle at toe-off compared to males. Ko et al. [19] investigated 174 males and 162 females aged 50–96 years at a self-selected walking speed and, similar to Boyer et al. [18], reported greater frontal plane hip ROM for the healthy females as compared to the males. However, in contrast to the latter [18], Ko et al. [19] reported reduced sagittal plane hip and no differences in knee kinematics for healthy females compared to males. Therefore, further research into potential gender-related differences in both knee OA and healthy, non-OA older adults is needed.

The first purpose of this study was to examine gender differences in gait kinematics at the ankle, knee and hip joints as well as foot and pelvis segments, in three planes of motion, for healthy individuals and individuals with mild-to-moderate knee OA. The second purpose of this study was to assess differences in gait kinematics between healthy gender-matched subjects as compared with their knee OA counterparts.

## Methods

### Participants

One hundred subjects with knee OA (males: *n* = 45; females: *n* = 55) participated in this study. The subjects ranged in age from 33 to 72 years. Their mean age, height, mass, body mass index (BMI) and walking speed are shown in Table 1. All the subjects with knee OA were categorized as being normal weight (18 ≤ BMI ≤ 25: *n* = 31), overweight (25 < BMI ≤ 30: *n* = 39), obese (30 < BMI ≤ 40: *n* = 27), or severely obese (BMI > 40: *n* = 3) [7]. Participants had symptomatic unilateral (left-side: *n* = 43; right-side: *n* = 37) or bilateral (*n* = 20) knee OA. Participants were included in the OA group if they met the American College of Rheumatology clinical criteria for mild-to-moderate knee OA [20]. Additionally, the following inclusion and exclusion criteria were used to determine eligibility [21]:

**Table 1:** Anthropometric characteristics and walking speed of study population for male and female subjects with and without OA

	OA		Control		*P*-value			
	Male (*n* = 45)	Female (*n* = 55)	Male (*n* = 18)	Female (*n* = 25)	OA male and female	Control male and female	Control and OA male	Control and OA female
Age (years)	55.18 (7.54)	55.33 (7.26)	54.83 (10.33)	52.12 (9.39)	0.92	0.38	0.88	0.10
Height (cm)	177.28 (7.53)	164.12 (6.92)	178.31 (4.90)	163.84 (8.19)	**<0.01**	**<0.01**	0.60	0.87
Weight (kg)	88.75 (14.12)	73.97 (15.00)	83.51 (13.45)	64.18 (12.20)	**<0.01**	**<0.01**	0.18	**<0.01**
BMI (m^2^/kg)	28.29 (4.67)	27.42 (5.14)	26.22 (3.58)	23.85 (3.57)	0.38	**0.04**	0.10	**<0.01**
Speed (m/s)	1.134 (0.05)	1.146 (0.03)	1.156 (0.05)	1.159 (0.02)	0.08	0.76	0.10	0.05

Bold number indicates statistically significant difference between groups of interest (*p* < 0.05)

#### Inclusion criteria

1. Have recent posterioanterior or skyline radiographs confirming the presence of knee OA.
2. Have a Kellgren-Lawrence (K-L) grade < 3.
3. Have a 100-mm knee pain visual analog scale (VAS) score > 20 mm on most days of the previous week.
4. The ability to walk on a treadmill without the use of handrails.

#### Exclusion criteria

5. Are diagnosed with severe knee OA (K-L grade > 3).
6. Are currently undertaking physiotherapy or other conservative management practices, including corticosteroid injections.
7. Have taken oral corticosteroids or anti-inflammatories in the 24 h prior to testing.
8. Have undergone, or were scheduled to undergo, joint preservation surgery or total joint arthroplasty.
9. Have evidence of OA in any other weight bearing joint.
10. Have systemic arthritic conditions.

A group of 43 healthy subjects (males: *n* = 18; females: *n* = 25) who had not experienced any musculoskeletal injuries over the 6 months prior to the time of testing, and had no clinical signs or symptoms of knee OA, was used for comparison. The subjects ranged in age from 40 to 79 years (Table 1). Healthy subjects were considered to be at normal weight (*n* = 24), overweight (*n* = 15), or obese (*n* = 4) [7]. Control participants did not undergo radiographic examination but did not meet any of the non-radiographic American College of Rheumatology criteria.

### Ethics, consent and permissions

The University of Calgary Conjoint Health Research Ethics Board (CHREB) approved the collection of the data (reference REB15-0557). Prior to collecting the data, all participants provided their written informed consent to participate and to have their anonymous/de-identified data stored in a research database. Thus, no individual participant’s data could be re-identified.

### Data collection

**Fig. 1:**
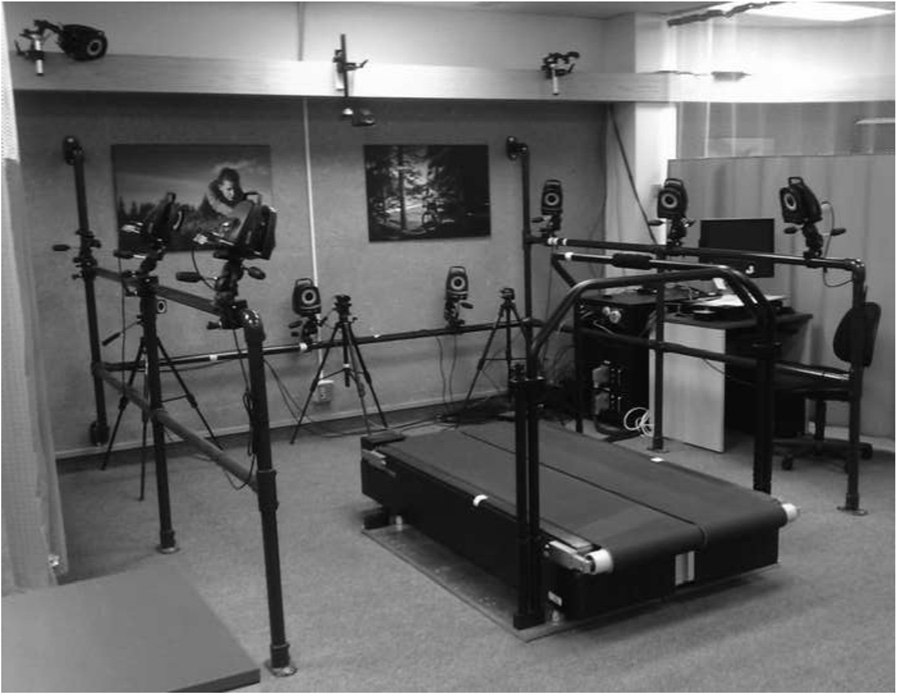
Photograph of the clinical laboratory used in this experiment

An 8-camera VICON motion capture system (MX3+, Vicon Motion Systems, Oxford, UK) and 9-mm retro-reflective markers, were used to collect 3-dimensional (3D) kinematic data at 120 Hz during untethered walking on a treadmill (Bertec Corporation, Columbus, OH). The lab set up is shown in Fig. 1. Markers were placed in the same manner described by Pohl et al. [22]. In brief, 14 anatomical markers were attached to the following landmarks: the greater trochanters, medial and lateral knee joint lines, medial and lateral malleoli, 1st metatarsal heads and 5th metatarsal heads bilaterally. Technical marker clusters, glued to a rigid plastic shell, were placed on the pelvis (three markers), and bilateral thigh and shank (four markers each) with self-adhering straps. Three markers were taped to the heel counter of each of the test shoes. These 25 markers represented seven rigid segments. Two markers individually placed on the anterior aspect of each shoe were used for used for detecting toe-off events. This marker set has been reported to produce highly reliable kinematic waveforms [22].

Following placement of all the anatomical and segment markers, the subject was asked to stand for a static trial. Standing position was controlled using a graphic template placed on the treadmill with their feet positioned 0.3 m apart and pointing straight ahead. Once the feet were placed in the standardized position, the subject was asked to cross their arms over their chest and stand still while one-second of marker location data were recorded to identify joint centre locations and to calculate the segment coordinate systems. Upon completion of the static trial, the 14 markers on the anatomical landmarks were removed. Walking kinematic data were collected while participants walked on a treadmill wearing standard shoes (Nike, Air Pegasus) for 30 s during which approximately 20–30 consecutive strides were collected for processing and analysis. Subjects were instructed to walk, without using the handrails, at a self-selected speed within a range of 1.0–1.3 m/s. After marker trajectories were filtered with a 10 Hz low-pass 2nd order recursive Butterworth filter, 3D rigid body kinematics were calculated using a single value decomposition approach outlined by Söderkvist and Wedin [23] and the joint coordinate system suggested by Cole et al. [24]. All participants were permitted as much time as they required to familiarize themselves with treadmill walking.

### Data processing

Kinematic joint angles during the gait cycle were calculated using 3D GAIT software (Gait Analysis Systems Inc., Calgary, Alberta, Canada), then segmented and normalized into 60 data points for the stance phase and 40 data points for the swing phase of the walking gait cycle (100 data points for one cycle). Stance phase was defined as initial heel contact to toe off, with initial contact identified as the point in time of the most anterior position of the superior calcaneal marker, and toe off was taken as the point in time of the most posterior position of the toe marker.

In examining differences amongst many variables between gender and diseased/injured groups, a significant challenge exists in lowering the dimensionality of the data in order to reduce the likely of Type I errors and overfitting. One way to accomplish this is to pre-select discrete variables, and due to strong correlations between angles of the same gait waveform (i.e., the same joint and plane of motion), a pattern of motion may be represented by only a few discrete angles of interest.

Consequently, eight discrete variables were selected for each waveform including: (1) angle at touchdown, (2–3) maximum and minimum peak angles during stance phase, (4) angle at toe-off, (5–6) maximum and minimum peak angles during swing phase, and (7–8) ROM angles during stance phase and swing phase. These discrete variables of interest were then averaged from ten consecutive strides of data to produce a mean for all three planes of motion for each of the three lower extremity joints (ankle, knee and hip), as well as the pelvis segment along with transverse- and sagittal-plane positions of the foot segment in the global coordinate system. These variables have been used in previous studies to investigate differences in gait kinematics between genders as well as between knee OA and control subjects [9]. Additionally, these variables have been used to effectively describe the key features of kinematic waveforms during the entire gait cycle [25].

Discrete variables were combined (8 discrete variables × {[(3 joints + 1 pelvis segment) × 3 planes] + [1-foot segment × 2 planes]} × 1 selected side) into one 112-dimensional row vector for each subject, creating an *n*-by-112 matrix used as an input for the analysis, where *n* is the number of subjects. These variables were extracted from the affected side for the subjects with unilateral knee OA and from the most affected side for the subjects with bilateral knee OA, while for the control subjects, discrete variables were randomly extracted from either left or right side.

### Data analysis

Data were analyzed across four groups: a male and female OA subject group (*n* = 100), a male and female healthy subject group (*n* = 43), a male OA and healthy male subject group (*n* = 63), and a female OA and healthy female subject group (*n* = 80). For each of the groups, two feature vectors were created based on the original discrete variables and a principal component analysis (PCA). First, the 112 discrete variables comprised the columns and the 100, 43, 63 and 80 subjects comprised the rows of the original feature matrix for the four groups above (*X*_100×112_, *X*_43×112_, *X*_63×112_ and *X*_80×112_), respectively. Second, to create the PCA feature matrix, the original feature matrix *X* was normalized such that columns of *X* were subtracted by the means and divided by the standard deviations. PCA is an orthogonal or a linear transformation technique used to convert a set of possibly correlated variables into a set of linearly uncorrelated variables by determining new bases (principal components or PCs) that maximize the variability in the original data [26]. The normalized matrix was transformed into the PC coefficients using the singular value decomposition (SVD) algorithm, which resulted in a coefficient matrix *V*_112×112_ for each of the normalized matrices. Similarly, PC variances or eigenvalues of the covariance matrix of *X*, (*L*_1×99_, *L*_1×42_, *L*_1×62_ and *L*_1×79_) were produced for each matrix. The PC scores (*Z*_100×99_, *Z*_43×42_, *Z*_63×62_ and *Z*_80×79_) were computed by multiplying the normalized matrix *X* by the PC coefficient matrix *V*, and used as the PCA feature matrix.

To examine the utility of the original discrete features and the PCA features in identifying and discriminating the differences between groups of interest, two approaches were used based on: (1) statistical criteria using univariate analyses, i.e., one-way analysis of variance (ANOVA) and Cohen’s *d* effect size, and (2) classification accuracy using a multivariate analysis, i.e., a support vector machine (SVM) classifier. Due to the use of multiple univariate statistical tests on multiple dependent variables, the resulting *p*-values from the ANOVA were controlled using a Holm-Bonferroni method [27] (i.e., adjusted *p*-value) for tests on all variables. Significant and meaningful features [28] were identified when *p* < 0.05 and *d* > 0.8. To examine classification rates, two models were used: (1) the original variable and (2) the PC scores [25, 29], as input for the linear SVM (with a soft margin parameter *c* of 1) [25, 30] to perform the classification separately. The number of original variables or PC scores was increased by 1 according to a descending order of effect size *d* at each step. The optimal number of original variables or PC scores was obtained when the maximum classification rate of SVM was produced according to the 10-fold cross validation method.

## Results

### Anthropometrics

A summary of the anthropometric and walking gait speed differences between control and OA males and females are shown in Table 1. Healthy and OA males were significantly taller and heavier than the females while healthy males and OA females had a higher BMI than the healthy females. OA females were also heavier as compared with the healthy females.

### Kinematic differences based on the statistical criteria

**Fig. 2:**
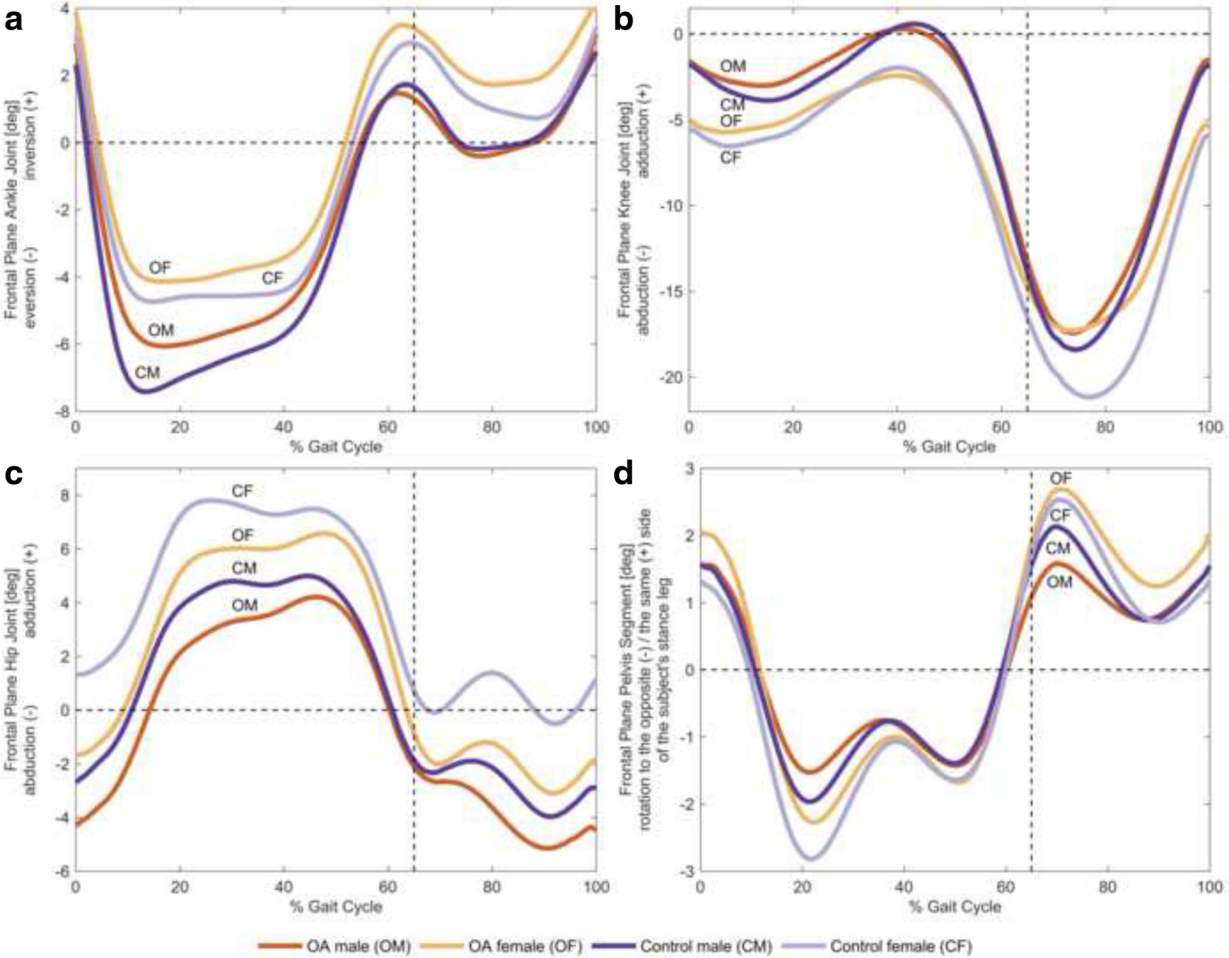
Frontal plane joint angles. The mean of individual time-normalized angles in the frontal plane for male and female subjects with and without knee OA during stance phase and swing phase. All angles are measured in terms of the distal segment relative to the proximal segment. **a** Ankle inversion and eversion, **b** knee adduction and abduction, **c** hip adduction and abduction, and **d** pelvis rotation to the same side and the opposite side of the subject’s stance leg

**Fig. 3:**
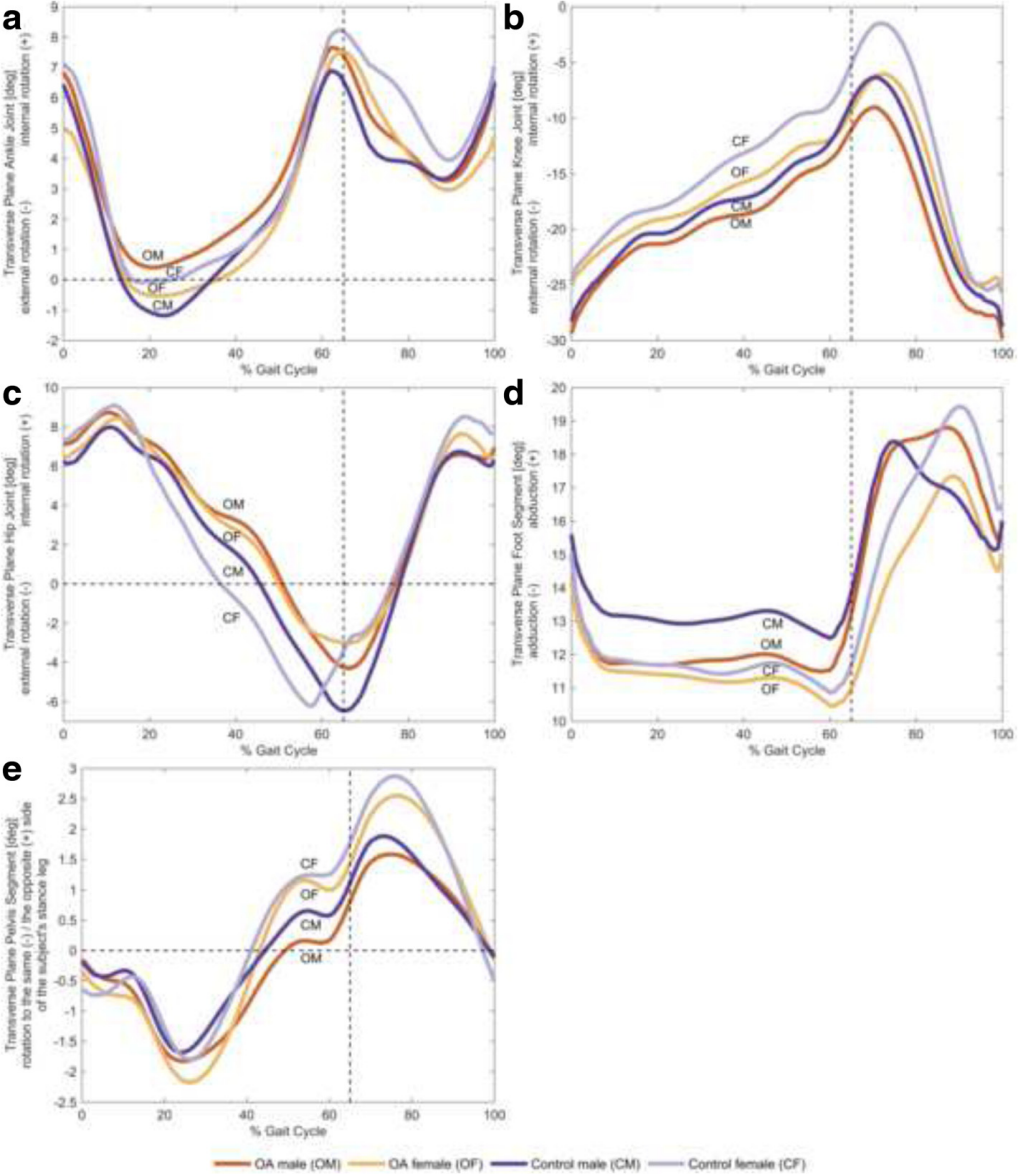
Transverse plane joint angles. The mean of individual time-normalized angles in the transverse plane for male and female subjects with and without knee OA during stance phase and swing phase. All angles are measured in terms of the distal segment relative to the proximal segment. **a** Ankle internal rotation and external rotation, **b** knee internal rotation and external rotation, **c** hip internal rotation and external rotation, **d** foot abduction and adduction, and **e** pelvis rotation the opposite side and the same side of the subject’s stance leg

**Fig. 4:**
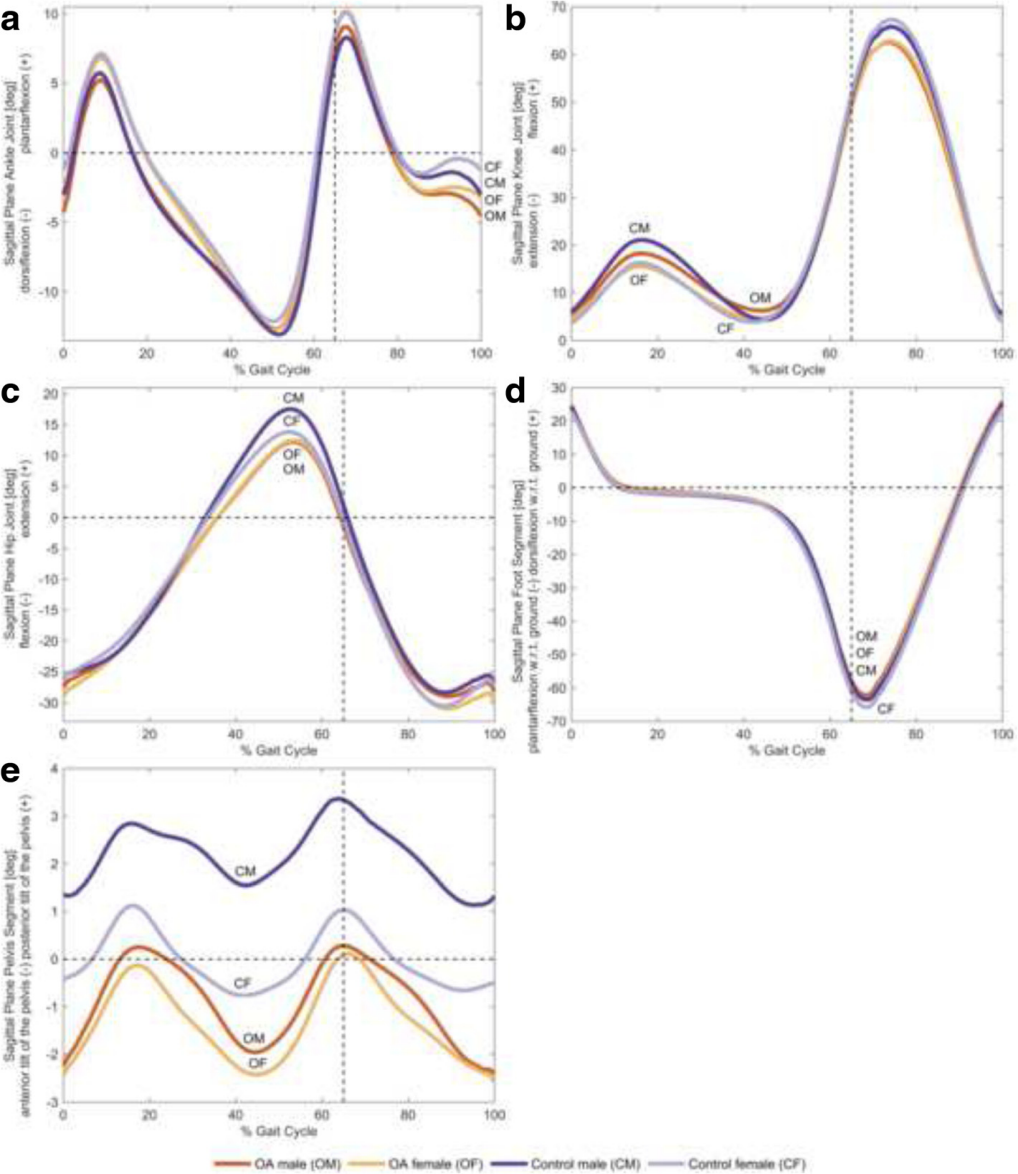
Sagittal plane joint angles. The mean of individual time-normalized angles in the sagittal plane for male and female subjects with and without knee OA during stance phase and swing phase. All angles are measured in terms of the distal segment relative to the proximal segment. **a** Ankle plantarflexion and dorsiflexion, **b** knee flexion and extension, **c** hip extension and flexion, **d** foot dorsiflexion and plantarflexion with respect to ground, and **e** posterior tilt and anterior tilt of the pelvis

The mean of the individual joint angles for each joint and plane of motion for each of the four gender-specific subgroups are presented in Figs. 2, 3 and 4. Of the 112 discrete variables of interest, statistically significant and meaningful differences between the 45 male and 55 female subjects with knee OA were found for three discrete variables (*p* < 0.05 and *d* > 0.8; Table 2). Specifically, OA females demonstrated greater knee abduction at touchdown and during swing, as well as a greater maximum peak hip adduction angle during stance as compared to OA males. For healthy subjects, the same statistically significant and meaningful differences were found between the 18 male and 25 female subjects without knee OA were found for three discrete variables (*p* < 0.05 and *d* > 0.8; Table 2). There were no significant differences between healthy males and OA males as well as between healthy females and OA females in the original discrete variables (*p* > 0.05).

**Table 2:** Comparisons of the discrete kinematic variables between male and female OA subjects (i.e., OM and OF) and between male and female healthy controls (i.e., CM and CF)

Joint	Plane of motion	Variable of interest	Mean angle (and its standard deviation) [deg]				Significant and meaningful difference: effect size *d*			
			OA male (OM)	OA female (OF)	Control male (CM)	Control female (CF)	Gender - OA (OM vs. OF)	Gender - Control (CM vs. CF)	Disease - Male (OM vs. CM)	Disease - Female (OF vs. CF)
Knee	Frontal	At touchdown	−1.37 (3.80)	−4.88 (4.06)	−2.02 (2.96)	−5.75 (2.91)	0.89*	1.27*	0.19	0.20
		Maximum peak during swing	−1.24 (3.86)	−4.66 (4.16)	−1.77 (2.89)	−5.20 (3.07)	0.85*	1.15*	0.16	0.15
Hip	Frontal	Maximum peak during stance	4.86 (3.73)	7.67 (3.21)	5.41 (1.94)	8.69 (2.61)	0.81*	1.43*	0.19	0.35

Bold number indicates a large effect size (*d* > 0.8)

*indicates statistically significant difference between groups of interest (*p* < 0.05)

### Kinematic differences based on the classification model

For gender differences in OA subjects, a maximum classification accuracy of 83 % was found between OA male and OA female patients using the top 19 ranked discrete variables sorted by effect size and the SVM classifier. Specifically, fifteen discrete variables (79 % of the feature vector) were extracted from frontal plane ankle, knee, hip and pelvis kinematics. The remaining variables (21 % of the feature vector) involved hip flexion-extension ROM angle during stance, the ROM of pelvic angles during stance and swing phases in transverse plane, and the knee external rotation angle at touchdown. When feature vectors were created based on the PC scores and then sorted by effect size, OA males and OA females could be separated with 99 % classification accuracy using a linear SVM ten-fold cross-validation method with the top 53 ranked PCs explaining 93.35 % of the variance in the data. It is important to note that useful information from all joints and planes of motion (i.e., all the 112 discrete variables) was extracted and contained in the PCA features.

For gender differences in healthy subjects, using the top 4 ranked discrete variables and the SVM classifier, the maximum classification accuracy of 86.05 % was found between healthy male and healthy female subjects. These variables were extracted from frontal plane knee kinematics (i.e., angles at touchdown and the maximum peak during swing) and hip kinematics (i.e., angles at the maximum and the minimum peaks during stance). Using the top 29 ranked PCs explaining 64.14 % of the variance in the data, healthy male and healthy female subjects could be separated with 100 % accuracy using the linear SVM classifier.

For the male subjects, the classification accuracy between healthy subjects and OA subjects was 80.95 % using the SVM classifier with the top 17 ranked discrete variables, and 100 % with the top 42 ranked PCs, explaining 65.45 % of the variance in the data. Specifically, thirteen discrete variables (76 % of the feature vector) were extracted from sagittal plane ankle, knee, hip and pelvis kinematics, while the remaining variables (24 % of the feature vector) were extracted from frontal plane pelvis kinematics.

For the female subjects, the classification accuracy between healthy and OA females was 71.25 % using the SVM classifier with the top 7 ranked discrete variables, and 98.75 % with the top 64 ranked PCs, explaining 91.95 % of the variance in the data. Specifically, six discrete variables were extracted from knee kinematics in all planes of motion. The other variable was the ROM of foot angles during the swing phase.

## Discussion

### Kinematic differences between OA males and OA females

The first purpose of this study was to examine gender-differences in gait kinematics between individuals with and without knee OA. The current study significantly builds upon previous research wherein the focus has been limited to only a single joint or plane of motion [5–8]. Moreover, the current investigation involved ankle, knee and hip joints, as well as foot and pelvis kinematics, for all three planes of motion, in an attempt to better understand the etiology of knee OA which is more prevalent in females as compared to males.

The results of the current study show that of the 112 discrete variables of interest, only kinematic variables for frontal plane knee and hip joint motion were significantly different between OA men and women during treadmill walking (Table 2). Specifically, OA females demonstrated greater knee abduction at touchdown and during swing as compared to OA males. These results are in contrast to Astephen Wilson et al. [5] who, using a similar PCA approach, reported that the only knee joint kinematic differences between OA males and OA females were in the sagittal plane knee angle range during stance. However, these authors [5] investigated severe knee OA patients prior to, and following total knee arthroplasty (TKA), and they only examined the differences in waveform shapes, not discrete variables, so comparisons with the current results are difficult. In the present study, OA females also exhibited significantly greater hip adduction angle at the maximum peak during stance in comparison to their male counterparts. Therefore, a novel finding of this study is that frontal plane hip and knee kinematics appear to be different between males and females, and the differences at the hip and the knee persist in both healthy and OA-symptomatic individuals.

In the sagittal plane, while McKean et al. [4] and Astephen Wilson et al. [5] both reported that females with moderate-to-severe knee OA exhibited reduced knee ROM angles across the gait cycle, similar results were not evident in the present investigation for subjects with mild-to-moderate knee OA. However, these findings are similar to Sims et al. [8] who also reported that knee ROM in OA females was no different compared to OA males with K-L grades of 1–4. It should be noted that the average walking speed observed by Sims et al., [8] (1.1 m/s) was more similar to that of the current study (1.1 m/s), contrary to McKean et al. [4] (1.3 m/s) and Astephen Wilson et al. [5] (0.9 m/s). The current results also suggest that the maximum peak knee flexion angle during the swing phase did not differ between OA males and OA females. This result is in contrast to Kaufman et al. [7], who observed this difference in 9 males and 11 females with OA. Therefore, based on the disparate findings amongst the current study and previous studies, further research may be necessary to better understand sagittal plane knee kinematics between OA males and OA females.

### Kinematic differences between healthy males and healthy females

The current study found overall agreement as compared to previous investigations involving gender differences in gait kinematics for older adults [18, 19, 31]. Specifically, in the current study healthy females exhibited significantly greater maximum peak hip adduction during the stance phase of gait as compared to healthy males (Table 2). These results are similar to most previous studies that have also reported differences in frontal plane hip joint angles between young, middle-aged and older healthy males and females during both walking and running [14, 15, 18, 19, 25, 31, 32]. It has been suggested that increased frontal plane hip motion, together with hip abductor muscle weakness, may be a factor related to why healthy females are more likely to experience a musculoskeletal injury such as patellofemoral pain [33] or iliotibial band syndrome [34], as compared with their male counterparts. In addition, these results are also in support to previous studies [15, 16, 31] wherein healthy females exhibited significantly greater knee abduction at touchdown in comparison to their male counterparts (Table 2).

In contrast to investigations involving younger and middle-aged healthy adults [14–16, 30], the current study found no significant differences in knee external rotation angles nor were there differences in hip internal rotation angles between healthy older males and females. A possible reason for these contradictory findings may be the subtle changes in gait associated with biological aging [30, 35]. The mean age of the subjects in the current study was 53.26 years, while the subjects in aforementioned studies were in their twenties or forties. Therefore, it appears that gender-specific gait kinematic differences for healthy older adults are dissimilar to those previously found for healthy younger adults.

### Kinematic differences between healthy subjects and OA subjects

The second purpose of this study was to assess the differences in gait kinematics between healthy gender-matched subjects as compared with their knee OA counterparts. There were no significant differences between healthy males and OA males or differences between healthy females and OA females in the original discrete variables. These results are partially agreement with the results of Ko et al. [11] and Weidow et al. [13] who reported no significant differences between healthy and OA subjects in knee kinematics for both gender-specific groups. On the other hand, these results are in contrast to a study by McKean et al. [4] who reported OA females exhibited less sagittal plane knee and ankle kinematics based on the PCA features of the gait waveforms as well as a study by Manetta et al. [10] who reported that OA males exhibited less knee flexion ROM during stance based on the discrete variables as compared to their healthy counterparts.

It is interesting to note that the standard deviations of the discrete variables, as well as the variability in waveform data, were both larger for OA affected males and females. This finding suggests an overall pattern of increasing variance, and possibly, individualized responses to disease progression, making characterization of the group as a whole, more challenging, especially when sample sizes are limited as in many of the aforementioned studies. Further research utilizing large sample sizes and sub-typing of OA individuals may provide valuable insight into characterizing gait changes in response to OA [36].

### Multivariate analysis and classification model

When the number of biomechanical variables is high and the between-group differences are relatively small, multivariate analysis and machine learning methods can provide insight into group biomechanical characteristics. This study clearly shows that a PCA and SVM approach can provide insight into complex relationships of biomechanical gait variables, as compared to multiple univariate analysis methods. However, this approach does have a trade-off in the interpretability of the result, as the feature vectors used to separate genders or disease states often include data from many different joints and planes. It is therefore advisable to combine both approaches for a more comprehensive understanding of biomechanical differences between groups.

To our knowledge, no previous investigations have utilized the PCA and SVM approach to discriminate between male and female subjects with and without knee OA during walking. Deluzio and Astephen [37] used a PCA and a linear discriminant analysis (LDA) approach to discriminate between healthy and knee OA mixed-gender groups and reported a classification accuracy of 92 %. The results of the current study show that classification accuracies of 98–100 % are possible for discrimination between males and females for both healthy and OA subject groups as well as between healthy and OA subjects for male and female subject groups using the PC scores as the input features for the SVM classifier. Although not as effective as the PCA approach, the original discrete variables still produced classification accuracies of 71–86 % when used as input features for the SVM classifier. Thus, careful consideration of the final interpretation of the data, as well as the desire for high classification accuracy, are both needed when deciding on a statistical approach.

### Limitations

Limitations to the current research study are acknowledged. We did not collect ground reaction force, or electromyography data and thus neither body kinetics, nor muscle activation patterns, were included in the analysis. However, Boyer et al. [18] reported no differences in the normalized ground reaction force between healthy and knee OA groups. Moreover, we chose to use joint kinematic angles to simplify the clinical interpretation of the results and shed some light on the greater prevalence of this disease in the female population as compared with males. Other clinical measures could also be incorporated to better understand the underlying mechanisms of knee OA. For example, future studies should include joint kinetics and ground reaction force data along with other clinical variables such as muscle strength, passive range of motion, muscle activation or knee stability to gain a greater understanding of sex-related differences in walking gait biomechanics, in an OA-affected population. In addition, self-reported pain and function scores, along with KL grade were only used for inclusion into the current study. Future studies should include these measures as previous studies have been shown to provide a better understanding gait kinematic patterns within distinct sub-groups of patients [38].

Since the subjects involved in the current study were all experiencing knee OA at the time of testing, and had been experiencing pain on most days of the previous week, cause and effect relationships cannot be established between the etiology of knee OA and walking biomechanics. However, the cross-sectional information gleaned from the current study has the potential to inform gender-specific rehabilitation and treatment approaches. Future prospective studies involving subjects, grouped by age and gender, will be an invaluable addition to the literature.

Confounding factors may exist between the groups studied, including pain (as previously mentioned), gait speed and BMI. We acknowledge that walking speed in individuals with knee OA may influence a number of gait biomechanical variables [39–41], however, the walking speeds of OA males (a mean of 1.134 m/s and a range of 1.01–1.23) and OA females (a mean of 1.146 m/s and a range of 1.06–1.21) in the present study were similar across groups and comparable to the self-selected normal walking speeds of a mixed-gender group of moderate knee OA patients in previous studies (e.g., a mean of 1.13 m/s and a range of 0.9–1.4 m/s [40]). BMI is also known to be an influential factor in the study of gait biomechanics [42, 43], and both hip and knee frontal plane kinematics in particular can be affected. There is, however, no consensus on the exact nature of these effects, and further research is needed to separate contributions of gender, BMI and joint disease to changes in overall gait.

## Conclusions

In conclusion, using discrete variables and a PCA approach, combined with an SVM classifier, the present study was able to accurately classify male and female subjects with and without knee OA as well as healthy and OA gender-matched groups. Although no differences in walking kinematic discrete variables were found in females and males with knee OA, in comparison to gender-matched healthy controls, subtle kinematic differences were still detectable by the non-linear multivariate classifiers. We therefore strongly recommended that future investigations involving knee OA patients and healthy controls be segmented according to gender and age. We also postulate that the lack of consensus amongst previous studies investigating the pathomechanics of patients with knee OA could be the result of mixed-gender cohorts.

### Ethics approval and consent to participate

The University of Calgary Conjoint Health Research Ethics Board (CHREB) approved the collection of the data (reference REB15-0557). Prior to collecting the data, all participants provided their written informed consent to participate and to have their anonymous/de-identified data stored in a research database. Thus, no individual participant’s data could be re-identified.

### Consent for publication

Not applicable.

### Availability of data and materials

The authors confirm that all data underlying the findings are fully available without restriction. All PCA data are available from the Running Injury Clinic and University of Calgary Institutional Data Access/Ethics Committee (CHREB) by contacting the corresponding author and Dr. Stacey A. Page, chair of CHREB at omb@ucalgary.ca.

## Abbreviations

3D: 3-dimensional
ANOVA: one-way analysis of variance
BMI: body mass index
K-L: Kellgren-Lawrence
LDA: linear discriminant analysis
OA: osteoarthritis
PCA: principal component analysis
ROM: range of motion
SVD: singular value decomposition
SVM: support vector machine
TKA: total knee arthroplasty
